# Supplementary material for: Robust Disease Prognosis via Diagnostic Knowledge Preservation: A Sequential Learning Approach
Source: medRxiv. 2025 Sep 25:2025.09.22.25336414. Preprint. [Version 1] doi: 10.1101/2025.09.22.25336414 (PMC12486016; doi:10.1101/2025.09.22.25336414)
Supplement: 1 [file NIHPP2025.09.22.25336414V1-supplement-1.pdf]

medRxiv preprint doi: https://doi.org/10.1101/2025.09.22.25336414; this version posted September 25, 2025. The copyright holder for this preprint (which was not certified by peer review) is the author/funder, who has granted medRxiv a license to display the preprint in perpetuity.

It is made available under a CC-BY 4.0 International license .

Supporting Information

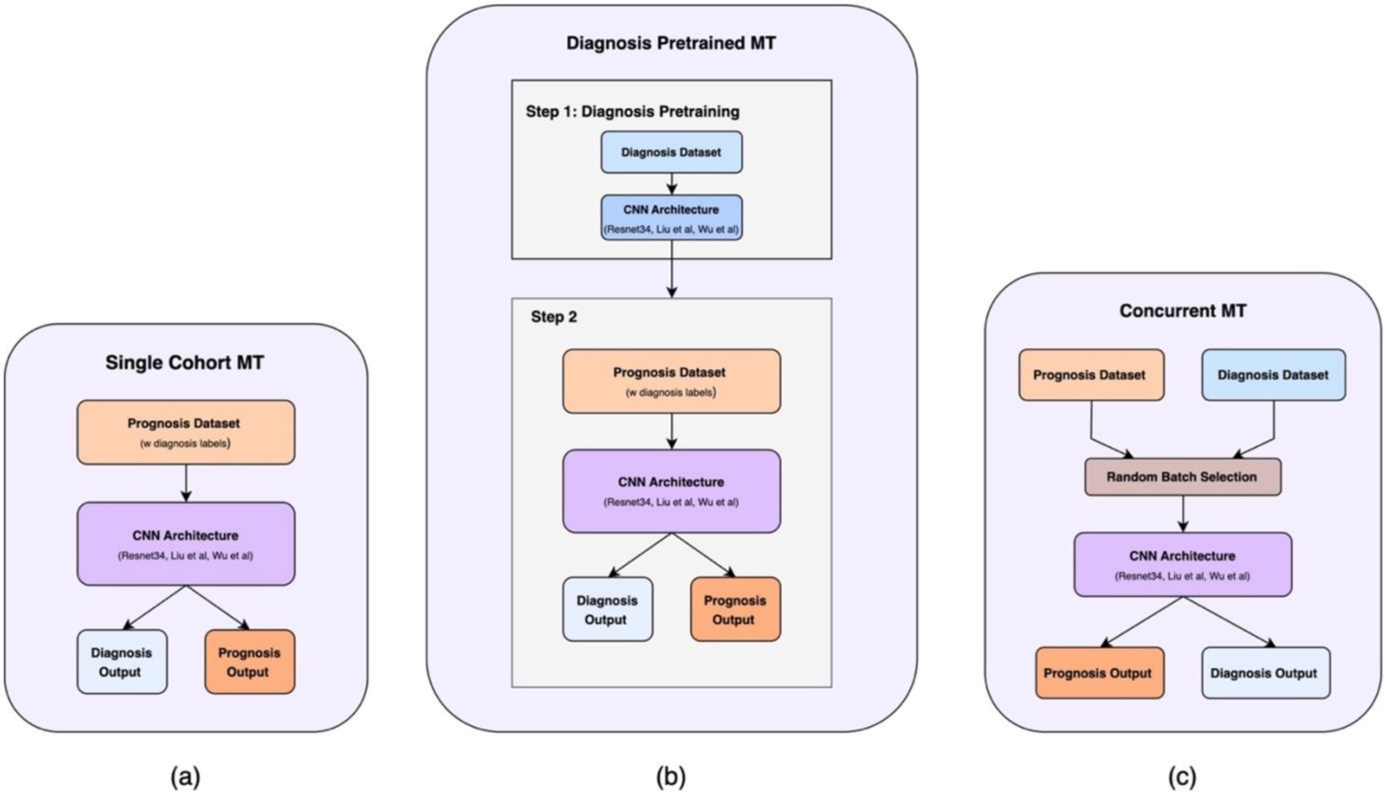

S1 Fig. Comparative multitask learning approaches. Visualizations of (a) Single Cohort MT, (b) Diagnosis Pretrained MT, and (c) Concurrent MT.

medRxiv preprint doi: https://doi.org/10.1101/2025.09.22.25336414; this version posted September 25, 2025. The copyright holder for this preprint (which was not certified by peer review) is the author/funder, who has granted medRxiv a license to display the preprint in perpetuity.

It is made available under a CC-BY 4.0 International license .

S1 Table. Patient Characteristics in Structural Progression OAI and MOST Cohorts.

Dataset	Parameters	Men		Women	
		**Patients**	**Controls**	**Patients**	**Controls**
**OAI Dataset**	No. of patients	216	707	355	970
	No. of scans	241	917	421	1348
	Mean age (y)	62.3±9.0	62.1±9.3	63.7±8.2	62.5±8.9
	Mean height (m)	1.8±0.1	1.8±0.1	1.6±0.1	1.6±0.1
	Mean weight (kg)	93.1±14.3	91.7±14.4	80.8±14.8	78.2±14.5
	Mean BMI (kg/m^2)	30.1±4.0	29.3±4.0	30.6±5.2	29.6±5.3
	**Ethnicity**				
	White	184	595	253	721
	Black	26	97	90	230
	Asian	2	4	3	5
	Other nonwhite	4	11	9	14
**MOST Dataset**	No. of patients	203	231	359	386
	No. of scans	231	282	427	495
	Mean age (y)	62.8±8.5	63.2±8.4	64.2±7.6	63.5±7.3
	Mean height (m)	1.8±0.1	1.8±0.1	1.6±0.1	1.6±0.1
	Mean weight (kg)	100.9±19.8	98.3±17.8	86.3±18.5	85.0±18.2
	Mean BMI (kg/m^2)	32.0±5.8	31.1±5.4	32.7±6.8	31.9±6.6
	**Ethnicity**				
	White	177	197	288	302
	Black	22	32	63	82
	Other	4	2	8	2

**Note: Mean data are presented as mean±standard deviation. BMI = Body Mass Index, MOST= Multi-Center Osteoarthritis Study, OAI= Osteoarthritis Initiative. Number of patients are shown for ethnicity data.*

medRxiv preprint doi: https://doi.org/10.1101/2025.09.22.25336414; this version posted September 25, 2025. The copyright holder for this preprint (which was not certified by peer review) is the author/funder, who has granted medRxiv a license to display the preprint in perpetuity.

It is made available under a CC-BY 4.0 International license .

S2 Table. Patient Characteristics in Structural Incidence OAI and MOST Cohorts

Dataset	Parameters	Men		Women	
		Patients	Controls	Patients	Controls
OAI Dataset	No. of patients	149	1103	271	1336
	No. of scans	160	1737	296	2171
	Mean age (y)	61.0±8.7	59.6±9.4	60.3±8.6	60.5±9.0
	Mean height (m)	1.8±0.1	1.8±0.1	1.6±0.1	1.6±0.1
	Mean weight (kg)	91.0±14.9	91.0±14.9	77.2±14.2	71.3±13.3
	Mean BMI (kg/m²)	29.1±4.1	28.1±3.8	29.3±4.8	27.1±4.8
	**Ethnicity**				
	White	133	975	208	1121
	Black	13	110	56	180
	Asian	1	4	4	13
	Other nonwhite	2	12	2	21
MOST Dataset	No. of patients	179	600	326	795
	No. of scans	196	945	371	1304
	Mean age (y)	61.3±7.8	60.6±7.8	62.4±8.0	61.1±7.7
	Mean height (m)	1.8±0.1	1.8±0.1	1.6±0.1	1.6±0.1
	Mean weight (kg)	99.8±17.1	93.7±15.1	82.6±15.6	77.4±14.1
	Mean BMI (kg/m²)	31.3±5.3	29.6±4.5	30.9±5.9	28.8±5.2
	**Ethnicity**				
	White	157	573	270	694
	Black	19	73	50	89
	Other	3	10	3	12

**Note: Mean data are presented as mean±standard deviation. BMI = Body Mass Index, MOST= Multi-Center Osteoarthritis Study, OAI= Osteoarthritis Initiative. Number of patients are shown for ethnicity data.*

medRxiv preprint doi: https://doi.org/10.1101/2025.09.22.25336414; this version posted September 25, 2025. The copyright holder for this preprint (which was not certified by peer review) is the author/funder, who has granted medRxiv a license to display the preprint in perpetuity.

It is made available under a CC-BY 4.0 International license .

S3 Table. Demographic Characteristics and Cognitive Status Distribution across the ADNI Prognosis Cohort.

**Parameters**	**Men**		**Women**	
	**Patients**	**Controls**	**Patients**	**Controls**
No. of patients	89	116	59	101
No. of scans	204	325	139	246
Age	75.7 ± 7.3	75.5 ± 6.2	73.5 ± 7.1	75.2 ± 5.9
Cognitive Normal (CN)	19	180	12	175
Mild Cognitive Impaired (MCI)	185	145	127	71
**Ethnicity**				
White	85	107	55	93
Black	2	5	3	8
Asian	2	4	1	0

Note: Data shows age (mean ± standard deviation) and number of subjects by cognitive status (CN vs MCI) for both controls (no disease progression, label = 0) and patients (Disease progression, label = 1).

medRxiv preprint doi: https://doi.org/10.1101/2025.09.22.25336414; this version posted September 25, 2025. The copyright holder for this preprint (which was not certified by peer review) is the author/funder, who has granted medRxiv a license to display the preprint in perpetuity.

It is made available under a CC-BY 4.0 International license .

S4 Table. Breast cancer Patient distribution in the Prognosis Cohort.

Parameters	Patients	Controls
No. of patients	2,319	2,837
No. of exams	3,000	3,000
Mean age (y)	61.5±11.4	56.4±10.8
**Exam-level BI-RADS**		
0	453	383
1	962	1,411
2	1,599	1,189
others	26	17
**Density**		
Almost entirely fatty	112	260
Scattered areas of fibroglandular density	821	1,200
Heterogeneously dense	1,119	1,124
Extremely dense	179	221
Unknown	88	32
**Ethnicity**		
White	1,588	1,774
Black	220	248
Asian	115	159
Other Nonwhite	396	656

medRxiv preprint doi: https://doi.org/10.1101/2025.09.22.25336414; this version posted September 25, 2025. The copyright holder for this preprint (which was not certified by peer review) is the author/funder, who has granted medRxiv a license to display the preprint in perpetuity.

It is made available under a CC-BY 4.0 International license .

S5 Table. Comparison of AUROC performance for models trained on progression and incidence, using different initializations.

Approach	Incidence (KL- 0,1)				Progression (KL - 2,3)			
	OAI AUROC	OAI AUPRC	MOST AUROC	MOST AUPRC	OAI AUROC	OAI AUPRC	MOST AUROC	MOST AUPRC
ImageNet pretrained	0.697 ± 0.02	0.222 ± 0.017	0.707 ± 0.019	0.380 ± 0.023	0.726 ± 0.011	0.561 ± 0.016	0.699 ± 0.011	0.672 ± 0.009
Diagnosis pretrained	0.699 ± 0.009	0.186 ± 0.011	0.742 ± 0.013	0.414 ± 0.027	0.718 ± 0.011	0.571 ± 0.015	0.731 ± 0.005	0.722 ± 0.009

S6 Table. Detailed AUROC analysis comparing model performance across cognitive status subgroups.

Approach	Within Group		Cross Group	
	CN (0 vs 1)	MCI (0 vs 1)	CN 1 vs MCI 0	CN 0 vs MCI 1
Random initialization	0.681 ± 0.103	0.750 ± 0.028	0.461 ± 0.065	0.888 ± 0.021
Diagnosis Pretrained	0.713 ± 0.037	0.773 ± 0.016	0.507 ± 0.029	0.924 ± 0.009

medRxiv preprint doi: https://doi.org/10.1101/2025.09.22.25336414; this version posted September 25, 2025. The copyright holder for this preprint (which was not certified by peer review) is the author/funder, who has granted medRxiv a license to display the preprint in perpetuity.

It is made available under a CC-BY 4.0 International license .

S7 Table. Detailed metrics for various training strategies for progression and incidence prediction tasks on OAI and MOST Datasets.

**Approach**	**Incidence (KL-0,1)**				**Progression (KL-2,3)**			
	**OAI AUROC**	**OAI AUPRC**	**MOST AUROC**	**MOST AUPRC**	**OAI AUROC**	**OAI AUPRC**	**MOST AUROC**	**MOST AUPRC**
Diagnosis pretrained Ref	0.700 ± 0.017	0.220 ± 0.019	0.751 ± 0.006	0.433 ± 0.008	0.725 ± 0.003	0.567 ± 0.008	0.735 ± 0.002	0.731 ± 0.003
Single Cohort MT	0.686 ± 0.026	0.223 ± 0.033	0.727 ± 0.048	0.406 ± 0.052	0.721 ± 0.013	0.552 ± 0.012	0.698 ± 0.010	0.663 ± 0.015
Concurrent MT	0.695 ± 0.021	0.242 ± 0.026	0.736 ± 0.011	0.405 ± 0.017	0.711 ± 0.014	0.532 ± 0.010	0.714 ± 0.013	0.702 ± 0.009
Diagnosis pretrained MT	0.704 ± 0.009	0.233 ± 0.016	0.739 ± 0.011	0.422 ± 0.018	0.724 ± 0.006	0.566 ± 0.008	0.709 ± 0.010	0.715 ± 0.007
Seq Learning w Replay	0.706 ± 0.012	0.209 ± 0.011	0.735 ± 0.007	0.397 ± 0.018	0.728 ± 0.013	0.570 ± 0.010	0.718 ± 0.016	0.713 ± 0.016

medRxiv preprint doi: https://doi.org/10.1101/2025.09.22.25336414; this version posted September 25, 2025. The copyright holder for this preprint (which was not certified by peer review) is the author/funder, who has granted medRxiv a license to display the preprint in perpetuity.

It is made available under a CC-BY 4.0 International license .

S8 Table. Detailed progression prediction performance (AUROC) across cognitive status subgroups for multitask methods.

**Approach**	**Within Group**		**Cross Group**	
	**CN (0 vs 1)**	**MCI (0 vs 1)**	**CN 1 vs MCI 0**	**CN 0 vs MCI 1**
Diagnosis pretrained Ref	0.713 ± 0.037	0.773 ± 0.016	0.507 ± 0.029	0.924 ± 0.009
Single cohort MT	0.618 ± 0.053	0.729 ± 0.020	0.418 ± 0.029	0.897 ± 0.024
Concurrent MT	0.733 ± 0.042	0.751 ± 0.014	0.506 ± 0.028	0.916 ± 0.021
Diagnosis pretrained MT	0.723 ± 0.038	0.777 ± 0.013	0.501 ± 0.030	0.938 ± 0.009
Seq learning w replay	0.735 ± 0.040	0.779 ± 0.007	0.514 ± 0.022	0.939 ± 0.012
